# Gene Expression Profiling Reveals Potential Players of Sex Determination and Asymmetrical Development in Chicken Embryo Gonads

**DOI:** 10.3390/ijms241914597

**Published:** 2023-09-27

**Authors:** Huaixi Luo, Hao Zhou, Shengyao Jiang, Chuan He, Ke Xu, Jinmei Ding, Jiajia Liu, Chao Qin, Kangchun Chen, Wenchuan Zhou, Liyuan Wang, Wenhao Yang, Wenqi Zhu, He Meng

**Affiliations:** Shanghai Key Laboratory of Veterinary Biotechnology, Department of Animal Science, School of Agriculture and Biology, Shanghai Jiao Tong University, Shanghai 200240, China; huangjingxiang@sjtu.edu.cn (H.L.); zhouhao1992@sjtu.edu.cn (H.Z.); jiangshengyao@sjtu.edu.cn (S.J.); mars-he@sjtu.edu.cn (C.H.); 22113033@whpu.edu.cn (K.X.); dingjinmei@sjtu.edu.cn (J.D.); jiajialiu@sjtu.edu.cn (J.L.); qin_chao@sjtu.edu.cn (C.Q.); chenkangchun@bsbii.cn (K.C.); zwc15639487225@163.com (W.Z.); kkkly88@sjtu.edu.cn (L.W.); yangwh202202@163.com (W.Y.); zwqjssz@sjtu.edu.cn (W.Z.)

**Keywords:** chicken, sex determination, sexual differentiation, high-throughput sequencing, transcriptome, asymmetrical development

## Abstract

Despite the notable progress made in recent years, the understanding of the genetic control of gonadal sex differentiation and asymmetrical ovariogenesis in chicken during embryonic development remains incomplete. This study aimed to identify potential key genes and speculate about the mechanisms associated with ovary and testis development via an analysis of the results of PacBio and Illumina transcriptome sequencing of embryonic chicken gonads at the initiation of sexual differentiation (E4.5, E5.5, and E6.5). PacBio sequencing detected 328 and 233 significantly up-regulated transcript isoforms in females and males at E4.5, respectively. Illumina sequencing detected 95, 296 and 445 DEGs at E4.5, E5.5, and E6.5, respectively. Moreover, both sexes showed asymmetrical expression in gonads, and more DEGs were detected on the left side. There were 12 DEGs involved in cell proliferation shared between males and females in the left gonads. GO analysis suggested that coagulation pathways may be involved in the degradation of the right gonad in females and that blood oxygen transport pathways may be involved in preventing the degradation of the right gonad in males. These results provide a comprehensive expression profile of chicken embryo gonads at the initiation of sexual differentiation, which can serve as a theoretical basis for further understanding the mechanism of bird sex determination and its evolutionary process.

## 1. Introduction

Chickens and most other birds are sex-determined genetically via sex chromosomes. Unlike mammals, which employ a XX/XY sex chromosome system and rely on the presence of the *SRY* gene (sex-determining region Y) located on the Y chromosome to determine maleness, chickens employ a different strategy, possessing a ZW sex chromosome system, where females carry two different sex chromosomes (ZW) and males carry two of the same (ZZ) [1]. There are two leading hypotheses for sex determination in chicken: W chromosome dominance and Z chromosome dosage [2,3]. The former denotes that W carries the female genes that determine ovarian development, whereas the latter posits that sex determination depends on the dosage of Z-linked gene expression which is higher in males [4,5]. Even though the two hypotheses have not been confirmed definitively yet, most studies supported the Z chromosome dosage effect.

During embryonic development, gonads undergo morphological differentiation to form testes or ovaries. In chicken embryos, genital ridges begin to be generated in the anterior region of mesonephros on embryonic day 3 (E3-Hamburger and Hamilton stage 18) [6,7], and separate from the mesonephros subsequently [7]. Genital ridges and mesonephros can be identified at E4.5 (HH 24) and separate from each other. At E6.5 (HH 29), genital ridges are more conspicuous and become gonads with the morphological characteristic of testes or ovaries, which suggests that sex begins to differentiate [7]. Furthermore, at this stage, several genes exhibiting sex-biased expression were identified, including *HINTW* [8], *FOXL2* [9,10], *CYP19A1* [11] and *WNT4*/*RSPO1* [12,13], which promote ovarian development in females, as well as *DMRT1* [14], *SOX9* [15], or *AMH* [16], which are associated with testis determination in males. The Z-linked gene DMRT1 is considered a key candidate for chicken testis determination. It can be detected as early as E3.5 and exhibited high expression levels in males [14,17], subsequently initiating the expression of genes involved androgen pathways such as *HEMGN* [18], *AMH* and *SOX9*. Ioannidis *et al*. [19] generated *DMRT1*-mutant chicken using CRISPR/Cas9, resulting in male chicken (ZZ) developing ovaries instand of testes, which proved that chicken sex fate is dependent on *DMRT1* dosage and involves the regulation of estrogen signaling. In female chicken embryos, in the absence of a substantial *DMRT1* expression, *FOXL2* begins to be expressed at E5.5 and subsequently activates *CYP19A1*, further promoting the estrogen synthesis pathway [11,12]. Another crucial gene promoting ovarian development is *Wnt4*, which stimulates the proliferation of follicular granulosa cells and also participate in chicken follicle selection [13]. Furthermore, Ayers et al. [20] conducted RNA-Seq to identify new sex-determination candidate genes, characterizing three female-biased genes, *CAPN5*, *GPR56*, and *FGFR3*, which potentially play significant roles in ovary development. However, the precise functional interactions among these genes are still unknown, and the existence of additional potential key candidates involved in sex determination remains uncertain.

Besides differential expression of sex-related genes, there are also distinctions in gonadal morphology between the two sexes. In contrast to mammals, which develop a pair of either testes or ovaries, most female birds only develop ovaries on the left side, with the right side undergoing degeneration, while males develop bilateral testes [21]. After sexual differentiation, in paired chicken testes, the testicular cords form in the medulla where Sertoli and Leydig’s cells differentiate and the cortical region degenerates. In contrast, in female chicken gonads, the cortical region of the left side proliferates and differentiates into the ovary, while the cortical region of the right side undergoes degeneration and ultimately vanishes. Several genes have been identified to be associated with chicken asymmetric ovarian development [22]. *PITX2* exhibits asymmetric expression in the female left cortex at an early stage (stage 18), which stimulates the proliferation of left cortex cells by regulating the expression levels of *RALDH2*, *Ad4BP*/*SF-1*, *ERα*, and cyclin D1 (stage 27) [23]. Additionally, even in symmetrically developed testes, differentially expressed genes can be detected, including *Nanog*, *PouV* and *Cvh*, which are related to the proliferation of primordial germ cells, (PGCs) [24]. On the subject of asymmetric or symmetric gonadal development, numerous studies have been primarily directed toward elucidatingthe mechanisms underlying the development of the female left gonad, with relatively less emphasis on the processes involving right gonad degeneration and the symmetrical growth of the male testes. Admittedly, a comprehensive understanding of all these mechanisms is yet to be fully elucidated.

In this study, we collected chicken embryo gonads during the critical periods of sex differentiation at E4.5, E5.5, and E6.5. Total RNA was extracted and subjected to sequencing using the next-generation sequencing technology Illumina and the third-generation sequencing technology PacBio. Subsequently, we conducted an analysis of gene expression profiles to identify differentially expressed genes associated with sex bias and asymmetrical gonadal development.

## 2. Results

### 2.1. Sex-Biased Transcript Expression Profile of Chicken Gonads at E4.5 Revealed via PacBio Sequencing

The total RNA of both sexes of chicken gonads at E4.5 was sequenced using the PacBio sequencing platform. We obtained 475,794 and 502,803 reads of inserts (RoI) for females and males, respectively. After clustering and merging, 13,178 and 14,813 collapsed isoforms for females and males were gained, respectively. Moreover, 99% of them were mapped to the genome. In total, 5593 genes for females and 6004 genes for males were annotated, and the total gene number was 6943 after merging. Additionally, 2334 and 2622 novel genes were unannotated for females and males, respectively (Table S1).

**Figure 1:**
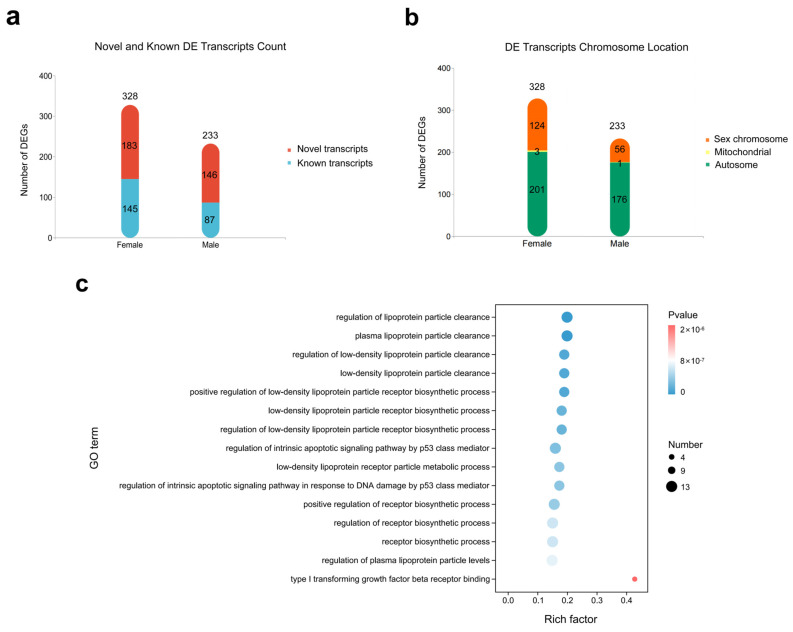
DE transcripts of male versus female at E4.5 from PacBio sequencing data. (**a**) Column chart of DE transcript number in males and females at E4.5. (**b**) Column chart of DE transcript chromosome in males and females at E4.5. (**c**) Top 15 GO enrichment terms of DEGs in females versus males at E4.5 (sort by *p*-value).

We identified 328 transcript isoforms that exhibited significant up-regulation in females, including 145 known isoforms and 183 novel isoforms. In contract, 233 transcript isoforms displayed male-biased expression, with 87 being known isoforms and 146 being novel isoforms (Figure 1a, Table S2). Transcript isoforms with high expression and foldchange levels are shown in Table 1. There were 38 differentially expressed genes (DEGs) expressed multiple different transcript isoforms, all displaying sex-specific biases. For instance, *EIF4G2*, *HNRNPK* and *SPTLC1* showed male bias, and each of them had three different differentially expressed (DE) transcript isoforms. In females, *HINTW*, *HNRNPKL*, and *HNRNPH2* also exhibited this type of expressive characteristics. Especially *HINTW* had 11 different DE transcript isoforms. Interestingly, 64 genes had up-regulated expression with different transcript isoforms in both sexes, including *HMGB3*, *IST1*, *USP10*, *HMMR*, etc. (Table S3). For example, *IST1* differentially expressed transcript ENSGALG00000000811 in males; meanwhile it expressed transcript ENSGALT00000086981 in females. The chromosome locations of DE transcript isoforms were also analyzed. Among the 561 DE transcript isoforms at E4.5, 180 transcript isoforms resided on the sex chromosomes (W: 62.8%; Z: 37.2%), 4 on mitochondria, and 337 on autosomes (Figure 1b). 

In total, 14,491 transcript isoforms were successfully annotated in the Gene Ontology (GO) database. 481 GO terms were significantly enriched (Table S4), which were mainly the synthesis and metabolism pathways of DNA, RNA, protein and cells (Figure 1c), including the regulation of lipoprotein particle clearance (GO:0010984), type I transforming growth factor beta receptor binding (GO:0034713), nuclear chromosome (GO:0000228) and so on. There were two GO terms related to sex, including female sex differentiation (GO:0046660), and sex chromosome (GO:0000803). In the female sex differentiation pathway, there were six DE transcripts, five of which were transcript isoforms of *HINTW*, suggesting that the gene may have an important influence on female gonadal differentiation.

### 2.2. Dynamic Changes in Gene Expression in Key Stages of Chicken Sex Differentiation

**Figure 2:**
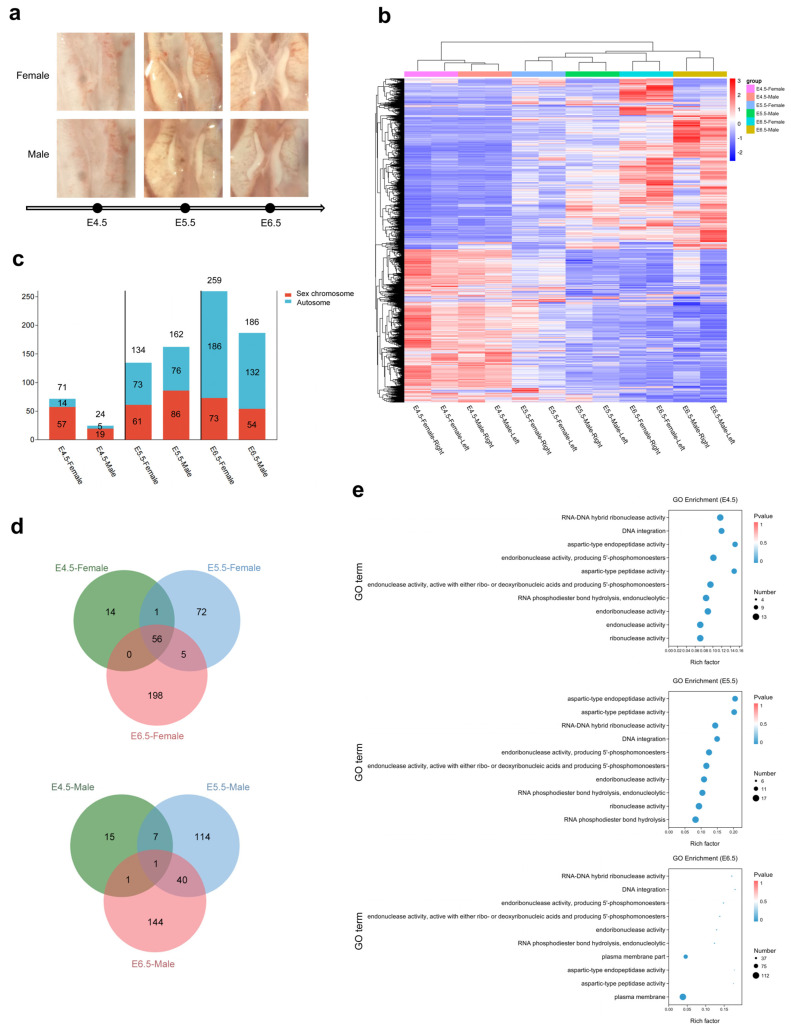
DEGs between males and females at E4.5, E5.5, and E6.5 from Illumina sequencing data. (**a**) Morphology of chicken embryonic gonads at the E4.5, E5.5 and E6.5 stages. (**b**) Heatmap illustrates the sample distances of 12 sample based on variable genes which are derived from the bilateral gonads of females and males at E4.5, E5.5, and E6.5. (**c**) Column chart of DEG counts and chromosomal localization in males versus females at the three stages. (**d**) Venn diagrams of the number of DEGs in males and females at the three stages. (**e**) Rich factor of top enriched 10 terms between males and females at the three stages (sort by *p*-value).

To investigate the gene expression profiles in key sex-differentiated stages and analyze the sex determination mechanism, we respectively collected and extracted RNA from 210 paired gonads at developmental stages E4.5 (female:male = 55:49), E5.5 (female:male = 30:25), and E6.5 (female:male = 31:20). The morphological characteristics of chicken embryonic gonads in these three stages are depicted in Figure 2a. Subsequently, we performed transcriptome seuqencing of chicken gonads using Illumina platform. After quality filtering, each sample yielded 40–49 million reads, with over 91% of these reads were mapped to the reference genome.

The dynamic changes in DEG counts across the three stages are illustrated in Figure 2c. Firstly, at E4.5, when the primitive gonads were isolated from mesonephros, 95 genes were significantly differentially expressed, comprising 71 up-regulated in females and 24 up-regulated in males (Table S5). Notably, the *HINTW* gene exhibited the highest expression levels in females, while *PRLR* (prolactin receptor) demonstrated the highest fold change in males. Secondly, at the later developed stage of E5.5, although sexual differentiation had not yet occurred, 296 genes showed significant differential expression between the two sexes, with 134 being up-regulated in females and 162 in males (Table S6). At this stage, certain important candidate genes associated with sex determination, such as *AMH* in males and *FOXL2* in females, began exhibiting significant differential expression. Thirdly, as gonadal differentiation initiated at E6.5, 445 genes displayed significant differential expression, 259 in females and 186 in males (Table S7). Among these, more known sex-determining genes exhibited differential expression, with some genes being expressed at higher expression levels than at E5.5, such as *SOX9* and *CYP19A1*, the latter of which exhibited a 1876-fold increase in expression (FPKM) compared to E5.5. In general, as the chicken embryos advanced in development, the total number of DEGs increased. At these three stages, the proportion of autosomal DEGs progressively rose, transitioning from 20% at E4.5 to 50% at E5.5, and peaking at 71% at E6.5 (Figure 2c). Notably, there were 57 DEGs overlapped across these three stages, with 56 of them being up-regulated female DEGs (55 genes resided on the W chromosome); only one gene, the *PRLR* gene, located on the Z chromosome, demonstrated male up-regulated expression. The counts of overlapped DEGs are showed in Figure 2d in Venn diagrams. Detailed dynamic changes of partial sex-biased gene expression are provided in Table 2.

Involved pathways of these DEGs were analyzed via topGO. The results revealed a significant enrichment of a total of 1051 GO terms. Specifically, at E4.5, E5.5, and E6.5, 205, 459, and 578 GO terms, respectively, exhibited significant enrichment (Tables S8–S10). The top 10 enriched GO terms of the three stages are shown in Figure 2e. Prior to the onset of gonadal differentiation (E4.5 and E5.5), the predominant pathways displaying significant enrichment were associated with biological process (BP) and molecular function (MF). These pathway primarily involved the binding and metabolism of DNA, RNA, and other molecules, along with certain peptidase activity pathways, including RNA–DNA hybrid ribonuclease activity (GO:0004523), DNA integration (GO:0015074), aspartic-type endopeptidase activity (GO:0004190) and so on. From E5.5 onwards, several pathways related to sex-specific processes showed significant enrichment, such as oocyte development (GO:0001555), somatic sex determination (GO:0018993), and female sex determination (GO:0030237). Notably, at the critical period of gonadal differentiation, E6.5, an increased number of cellular component (CC) categories appeared among the top 30 significantly enriched GO terms. Importantly, multiple sex-related pathways began to activate at this stage, including gonad development (GO:0008406), development of primary sexual characteristics (GO:0045137), and sex differentiation (GO:0007548). 

Illumina and PacBio sequencing platforms were used to conduct the sequencing of E4.5 chicken embryonic gonads. From Illumina sequencing data, a total of 95 DEGs with sex-biased expression were identified, while the PacBio sequencing results revealed 382 DEGs, encompassing 561 transcript isoforms. Within these DEGs, 21 were found to overlap in females, and 4 exhibited overlaps in males (Table S11). All of these overlapping DEGs were located in sex chromosome, except for *ASL1*, which was resided on Chromosome 19. Based on the Illumina sequencing results, it was observed that *HINTW* displayed the highest expression among theses overlapped DEGs in females, while *ZFAND5* exhibited the highest expression in males.

### 2.3. Left–Right Asymmetric Gene Expression in Embryonic Chicken Gonads

**Figure 3:**
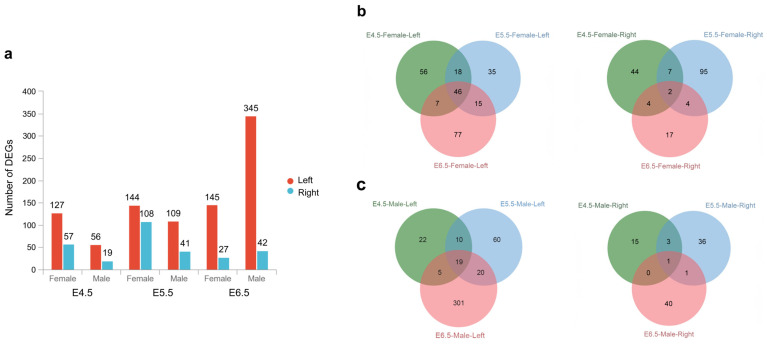
DEGs of left versus right gonad in males and females at E4.5, E5.5, and E6.5, revealed by Illumina sequencing. (**a**) Column chart of DEGs number of left versus right in female and male gonads at the three stages. (**b**,**c**) Venn diagrams of the number of DEGs in female (**b**) or male (**c**) left and right gonads for each stage.

To investigate asymmetric gene expression profiles in the left and right chicken embryonic gonads, we conducted comparative analysis of Illumina sequencing results obtained from both sides of gonads. The number of DEGs and their patterns of change are showed in Figure 3a, and comprehensive information on all DEG in both left and right gonads is provided in Tables S12–S17. Our sequencing results revealed a notable trend: there were more up-regulated genes in the left gonads compared to the right gonads in both sexes. Furthermore, the number of DEGs increased with the duration of incubation.

At E4.5, a comparison of the left and right gonads in females revealed the presence of 184 DGEs.Notably, DEGs with relatively higher expression (FPKM > 100) in the left gonad included *GJA1*, *LTBP1*, *MSC*, and *PITX2*. These genes encoded proteins associated with gap junctions for intercellular channels, extracellular matrix protein [25], muscle growth and cell differentiation/proliferation, respectively. These DEGs are directly or indirectly involved in processes of cell proliferation and differentiation, suggesting that from E4.5, female gonads are prepared for asymmetric development. Regarding males at E4.5, a total of 75 DEGs were identified when comparing the left and right gonads, which was over 100 DEGs less than the number of those in females, indicating a lower degree of asymmetrical gene expression in the male left and right gonads. Surprisingly, DEGs with relatively higher expression in male left gonads also included *GJA1*, *MSC*, and *PITX2*. At E5.5, a total of 222 DGEs were identified in the left and right gonads of females. Among the top 5 DEGs with the highest expression levels (FPKM > 100) in left gonads included *MSC*, *PIP5K1B*, *DAZL*, *NKAIN4* and *CLDN1*. Notably, *PIP5K1B* and *DAZL* are associated with the regulation of several cellular processes or germ cell growth. *NKAIN4* and *CLDN1* are related to the maintenance of the basic energy and protein required for cell growth. In the right gonad, one of the highly expressed DEGs was *SLC25A37*, which is involved in the negative regulation of angiogenesis and blood vessel endothelial cell migration. This indicated that these DEGs that collectively promote cell development and concurrently exert a negative regulatory influence on angiogenesis, thereby might contribute to the asymmetric development of female gonads. With regard to male left gonads at E5.5, a total of 151 DEGs were identified, with *PIP5K1B* exhibiting the highest expression levels among the DEGs at this developmental stage. At E6.5, there was a slight reduction in the number of DEGs, with a total of 173 DEGs identified. Meanwhile, the DEG count of the male left and right gonads dramatically increased to 388. Especially, *HBA1*, *HBAD*, *HBBR* and *HBZ* exhibited extremely high expression levels (FPKM > 4000). Furthermore, we observed that 46 DEGs in the left gonads exhibited overlapping expression across all three developmental stages in females, whereas only 2 exhibited overlap on the right side. In males, 19 DEGs in the left gonad displayed overlapping expression across these stages, while the right side had only 1 such DEG (Figure 3b,c, Table S18). Surprisingly, among these left gonad-expressing overlapping genes, 12 DEGs existed in both sexes, including *PITX2*, *SCAPER*, *PALMD*, *LUZP2*, *SLC1A3*, *GDF8*, *LOC422926*, *GRIA1*, *GPR157*, *CNTN2*, *PDC*, and ENSGALG00000001894, which were involved in cell proliferation and differentiation, cellular developmental process and other pathways. This observation suggests that similar asymmetric developmental pathways are engaged in both males and female gonads.

GO enrichment analysis was conducted to elucidate the pathways involved in the asymmetric development of chicken gonads (Tables S19–S24). In females, a total of 1806 pathways displayed significant enrichment across the three developmental stages. Among these GO terms, 939 showed significant enrichment at E4.5. Within the top 30 enriched GO terms at this stage, there were fibrinolysis (GO:0042730), blood coagulation (GO:0007596), hemostasis (GO:0007599) and 13 other pathways associated with blood. Additionally, it’s noteworthy that the involved DEGs exhibited up-regulation in the right gonads of females. At E5.5, 743 pathways showed significant enrichment, primarily encompassing processes related to DNA modification, renal tissue formation, cell proliferation, and apoptosis. Meanwhile, at E6.5, 627 pathways showed significant enrichment, and a certain number of DEGs were involved in cell proliferation and differentiation, molecular transport, and DNA modification. Specifically, these processes included multicellular organism reproduction (GO:0032504), cell differentiation (GO:0030154), DNA methylation or demethylation (GO:0044728). In contrast, pathways related to blood coagulation, hemostasis, fibrinolysis, and similar processes showed up-regulation in the right gonads of females, while pathways associated with cell proliferation and differentiation were up-regulated in the left gonads of females. Notably, one of the overlapping DEGs across all three stages in the female right gonads, *P2RY8* (*purinergic receptor P2Y8*), was found to be involved in blood coagulation and hemostasis pathways, suggesting that the apoptosis of right gonadal cells may have commenced at E4.5 and due to potential disruption of blood circulation. In males, a total of 1655 pathways were significantly enriched at the three stages. Among these pathways, 694 demonstrated significant enrichment at E4.5, while 450 pathways displayed significant enrichment at E5.5. At the two gonadal undifferentiated stages, DEGs were primarily involved in cellular communication and signaling pathways, tissue/cell morphogenesis, molecular regulations, *etc*. These pathways included cell-cell signaling (GO:0007267), cell communication (GO:0007154), blood vessel morphogenesis (GO:0048514), node of Ranvier (GO:0033268) and so on. Until the gonadal differentiation stage (E6.5), several pathways related to blood oxygen transport/binding, cell proliferation or differentiation, and signal transmission began to show significant enrichment. This included pathways such as hemoglobin complex (GO:0005833), oxygen transport (GO:0015671), cell morphogenesis (GO:0000902). A noteworthy discovery among the overlapping DEGs across all three stages in the left gonad of males was the highest expression of *PIP5K1B*, a gene previously linked to spermatogenesis in male mice. Previous research showed that *PIP5K1B*-knockout male mice exhibited sterile, confirming its role in spermatogenesis [26]. Hence, we speculate that asymmetric development also occurs in male gonads, with the right side potentially increasing blood oxygen levels to improve blood circulation and prevent degeneration, contrasting the situation observed in females.

### 2.4. Validation of Differentially Expressed Genes by qPCR

**Figure 4:**
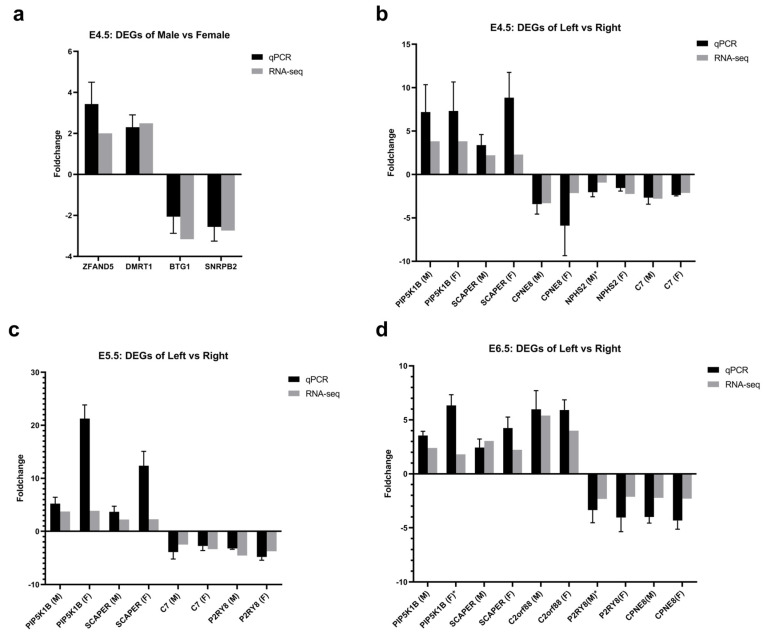
Verification of 11 DEGs from males versus females and left versus right via qRT-PCR, compared with the expression levels obtained via RNA-seq. qRT-PCR values were normalized relative to the expression levels of β-actin in the same cDNA sample. (**a**) DEGs of male and female gonads at E4.5 from PacBio sequencing. Expression data are presented as expression values of genes in male gonads relative to those in female samples. (**b**–**d**) DEGs of left and right gonads at E4.5, E5.5 and E6.5 from Illumina sequencing. Expression data are presented as expression values of genes in the left gonads relative to those in the right samples. Data are expressed as the mean ± SEM of three biological replicates (* the genes marked with an asterisk are not significantly differentially expressed in the RNA-seq data of this group.)

To validate the expression levels, we used qPCR to quantitate the expression of 11 DEGs spanning from E4.5 to E6.5. These DEGs comprised 4 genes identified as DEGs in males and females via PacBio sequencing and 7 genes distinguished as DEGs in the left and right via Illumina sequencing. These genes included known and potential candidate genes. *ZFAND5* and *DMRT1* were male up-regulated DEGs; *BTG1* and *SNRPB2* were female up-regulated DEGs; *PIP5K1B*, *SCAPER* and *C2orf88* were left gonadal up-regulated DEGs; *CPNE8*, *C7*, *NPHS2* and *P2RY8* were right gonadal up-regulated genes. The qRT-PCR results were consistent with the sequencing results (Figure 4). Although some discrepancies between the qPCR and RNA-seq results were noted, they remained within acceptable limits to satisfy the foldchange criteria for selecting DEGs with |log_2_ foldchange| > 1.

## 3. Discussions

### 3.1. Sex Determination and Gonadal Differentiation-Related Genes in Chicken Embryos

This study conducted transcriptome sequencing using both Illumina and PacBio platforms to analyze gene expression patterns in chicken embryonic gonads during early developmental stages. The identification of sex-related DEGs in this study aligns with previous RNA-seq studies during the critical period of chicken sex determination. For instance, we found that highly differential expression of *HINTW* in females at E4.5, an emerging female bias in *FOXL2* at E5.5, and a remarkable 1896-fold increase in the expression of *CYP19A1* at E6.5 compared to it levels at E5.5. Similarly, known male candidate genes such as *DMRT1* showed significant differences at E4.5, while *AMH* and *LHX8* showed these differences at E5.5, and *SOX9* showed this male bias at E6.5 [2]. Moreover, our observations indicate that both the number and expression levels of DEGs increased with the duration of incubation, and the cumulative number of DEGs in females at three stages exceeded that observed in males. Estermann *et al*. [27] reported findings from single-cell sequencing of chicken embryonic gonads, along with cellular localization analysis, identifying *PAX2* as a marker for undifferentiated supporting cell progenitors in chickens. These *PAX2*-positive cells migrated from the mesonephros to the gonads. In our sequencing results, we observed a significant up-regulation of *PAX2* in female gonads at E6.5, suggesting a potentially greater presence of supporting cell progenitors in female gonads in comparision to male gonads. The substantial up-regulation of female-biased genes may consequently steer these cells toward a female development fate.

At the early stage of gonadal differentiation, a substantial proportion of DEGs were associated with W- and Z-linked genes. However, this proportion gradually decreased as gonadal development progressed. This trend suggests that early sex determination is influenced by differential expression of sex-linked genes. In addition, significant differential expression was observed in certain mitochondrial genes. This finding implies that W-linked genes may play a more important role before gonadal differentiation, while mitochondrial genes also contribute to the process of sex determination. Furthermore, autosomal genes may influence sex determination by transcribing different isoforms. Regarding enriched GO terms in the three stages, these terms were primarily associated with pathways related to binding and matabolism of DNA, RNA, and other molecule, as well as certain peptidase activities. Furthermore, pathways relevant to sex determination, such as oocyte development and female sex determination, exhibited significant enrichment at E5.5 and E6.5.

Notably, the full-length sequencing results of E4.5 gonads displayed that a significant portion of the up-regulated transcripts in females originated from the W chromosome, especially, certain genes expressed multiple transcript isoforms. For instance, *HINTW* transcribed 12 isoforms and *HNRNPKL* had 9, several of which were new transcripts isoforms. The abundance of gene expression was evident in the number of up-regulated transcript isoforms, a feature not easily discernible through the second-generation sequencing. Furthermore, we also found that 64 genes, including *HMGB3*, *IST1*, *USP10*, and *HMMR*, exhibiting high expression levels and high fold change with different transcript isoforms in males and females. Most of these genes are located on autosomes, while only six genes, namely *PRKAA1*, *ISCA1*, *REEP5*, *RIOK2*, *CETN3*, and *DHFR*, are on the Z chromosome. For instance, *HMGB3*, which was the most highly expressed gene among the 64 genes, differentially transcribed isoform ENSGALT00000014763 in males and isoform ENSGALG00000009071 in females. *HMGB3* plays a key role in DNA repair, recombination, transcription and replication [28], as well as regulation cell proliferation in mammals via increasing or decreasing the expression of *NANOG* and *SOX2* [29]. In addition, sex chromosome GO:0000803 was one of the significantly enriched pathways The associated gene *UBE2B* (ubiquitin-conjugating enzyme E2B) expressed two different transcript isoforms in both sexes. *UBE2B* encodes a ubiquitin-binding enzyme highly conserved in eukaryotes, and it is essential for DNA damage repair after replication. *UBE2B* has been validated to be implicated in spermatogenesis in male mice [30], suggesting that *UBE2B* potentially has a repair effect on sex chromosome DNA replication and contribute to chicken gonadal development. Previous studies primarily focused more on the effect of sex chromosome genes in initiating sex differentiation [31,32,33]. However, the third-generation sequencing results indicate that autosomal genes may also play an important role in gonadal differentiation and sex determination by expressing different transcript isoforms in males or females, respectively. 

Although numerous studies have demonstrated that the Z-linked gene *DMRT1* is the key sex-determining gene dominating male sex differentiation [34], interestingly, we found that *PRLR* was the only overlapped DEG in the three stages in males. The *PRLR* gene, which is conserved in various species including mice, zebrafishes, dogs and humans, encodes the receptor of prolactin, an anterior pituitary peptide hormone essential for reproduction. The *PRLR*-knockout female mice displayed lactation failure or multiple reproductive abnormalities [35]. In chickens, *PRLR* is located on the Z chromosome and has been proven to be associated with broodiness and egg production, making it a potential molecular marker for chicken breeding [36]. However, it has not been proven that the up-regulation of *PRLR* in male chicken embryos is related to sex determination. Moreover, our results suggest that female pathways exhibit more significant differential activity, with a higher number of DEGs, especially marked by a high expression of the W-linked gene *HINTW. HINTW* is expressed in multiple tissues of female chicken embryos [8] and has been considered to be the most promising candidate gene for female sex determination formerly. However, Smith *et al*. [37] reported that overexpression of *HINTW* gene via retrovirus in chicken embryos did not reverse the sex of male embryos and had no influence on the normal development of female embryos. They also proposed that the protein encoded by *HINTZ*, a Z chromosome-homologous gene, could form a dimer with *HITNW* in vitro, thereby reducing the role of HINTZ in promoting testicular development [37,38]. In addition, they performed antibody tests for HINTW-predictive protein but did not detect endogenous HINTW protein, suggesting that it may have been transcribed but not translated. 

### 3.2. Left–Right Asymmetric Development of Chicken Gonads 

Before the onset of sex differentiation, the gonadal morphology in both sexes is similar, and slight differences between male and female can be observed after E6–6.5. Primitive gonads contain two distinct layers, the cortex and medulla [3]. Witschi [39] proposed the asymmetric distribution of PGCs in chicken gonads in 1935. During the process of sexual gland differentiation, the medullary region of the testes develops into testicular cords containing male primordial germ cells (PGCs), within which Sertoli cells proliferate. Simultaneously, in the interstitial tissue surrounding the cords, Leydig cells develop, responsible for the production of male hormones. Besides, the development of the cortex is relatively weaker compared to the medullary region. In contrast, ovarian development involves the thickening of the cortex, with female PGCs predominantly concentrated in the cortical region, where they undergo proliferation and differentiation. Due to the asymmetric expression of *PITX2*, the distribution of retinoic acid (RA) becomes uneven, resulting in a lack of meiotic division and subsequent apoptosis in PGCs of the left medullary and right gonadal regions following hatching. In our sequencing data, we found the asymmetrical expression of genes consistent with the asymmetrical development of gonads. However, both female and male left gonads differentially expressed plentiful genes, including *PITX2*. The *PITX2* gene is conserved in multiple species and is involved in the asymmetric expression of the left-right axis. *PITX2*-knockout mice developed organ-asymmetric abnormalities [40]. *PITX2* is highly expressed in the female left gonadal cortex and up-regulates *RALDH2* (RA-synthesizing retinaldehyde dehydrogenases 2), ERα and cyclin D1 to stimulate cell proliferation [23]. Previous studies have indicated that the overexpression of *PITX2* in the right gonad can induce cortical differentiation and prevent degeneration [41]. Therefore, it is plausible to suggest that *PITX2* may be the key gene that induces the asymmetric development of chicken ovaries. Estermann *et al*. [27] also reported in their study that *CYP17A1* serves as a steroidogenic marker and belongs to the theca cell lineage genes which possess the capability to synthesize substrates for androgens. In our research, we observed a significant up-regulation of *CYP17A1* in male left gonads at E6.5, suggesting a potentially higher abundance of male left gonadal germ cells compared to those in the right gonads. Their study also mentioned *DAZL* as a marker for germ cells primarily localized within the cortical area. In females, the left gonadal cortex undergoes development while the right gonadal cortex regresses. Our sequencing results consistently demonstrated an up-regulated expression of the *DAZL* gene in the left gonads of females at E4.5, E5.5, and E6.5, aligning with previous research findings.

**Figure 5:**
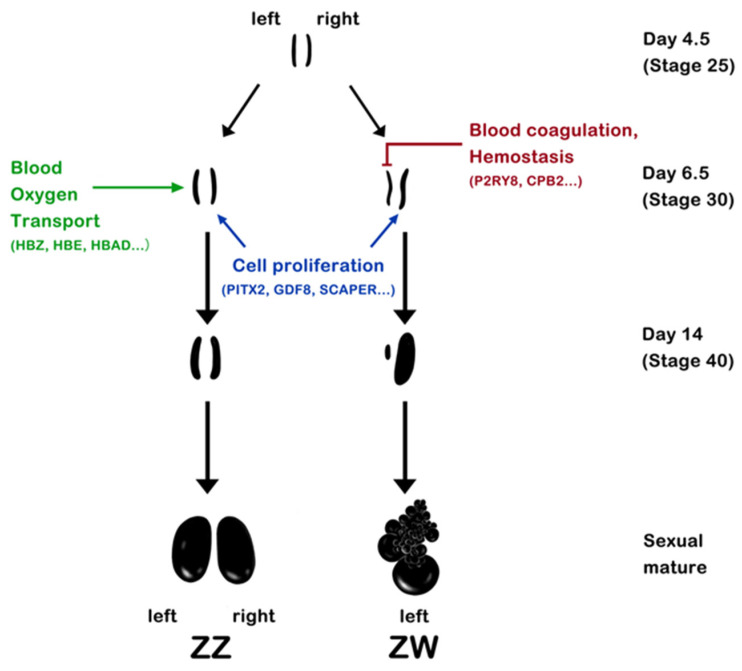
A putative mechanism of gonadal asymmetrical development in male and female chicken.

While most studies have primarily focused on elucidating the mechanisms behind the asymmetric development of the female gonads [42,43], comparatively less attention has been given to understanding the symmetric development of the male testes. In our analysis, we analyzed the expression profiles of the left and right gonads in chicken embryos at E4.5, E5.5, and E6.5 via Illumina sequencing. Our results consistently revealed that at all three developmental stages, there were more DEGs in the left gonads of both sexes compared to the right gonads. Notably, we identified 12 overlapping DEGs of the left gonads in both males and females, including *PITX2*, *GDF8*, *LOC422926*, etc. This observation suggested that both male and female gonads developed left-right asymmetry in the early stages, potentially sharing similar underlying mechanisms. However, several genes exhibited significantly higher expression and up-regulated in male right gonads at E6.5, which were related to blood oxygen transport, such as *HBA1*, *HBAD*, *HBZ* and *HBBR*. These genes were highly expressed in both male and female gonads at E4.5 and E5.5, with no significant differences between the left and right gonads, and their expression levels decreased as incubation progressed. Nevertheless, these genes were up-regulated in the male right gonad at E6.5, accompanied by a notable increase in fold change, suggesting that male chicken embryos may employ these blood oxygen transport pathways in the right gonads to prevent degeneration as they continue to develop. We have generated a hypothetical pathway diagram depicting the putative mechanism of asymmetric development in chicken embryos based on the DEGs analysis and enriched pathways in our study (Figure 5). Besides, sex-determining candidate genes may also be involved in symmetric or asymmetric gonadal development. Ioannidis *et al*. [19] conducted a targeted knockout of *DMRT1* on one of the Z chromosomes in male chicken embryos, resulting in sexual reversal of the gonads. The edited male chicken (Z^DMRT1+^Z^DMRT1−^) exhibited ovarian morphology, where the right side underwent degeneration, while the left side developed normally. Although this study did not explore the phenomenon of gonadal asymmetric development after sex reversal, the results suggested that male chickens without one allele of *DMRT1* did not develop symmetrical ovaries. This indicated that *DMRT1*, and potentially other male development genes, may also be involved in testicular symmetrical development.

## 4. Materials and Methods

### 4.1. Chicken Egg Incubation and Gonad Sampling

Specific pathogen-free (SPF) fertilized White Leghorns eggs (Sais Poultry, Jinan, China) were incubated at 60–65% humidity and 37.8 °C in an OVA-Easy 380 incubator (Brinsea, North Somerset, UK) until E4.5, E5.5, and E6.5. Under a microscope, the right and left gonads of the embryos at each stage were collected with sterilized microsurgical tweezers and scissors, and placed into 1.5 mL RNase-free centrifugal tubes individually. We collected 104, 55, and 51 paired gonads at E4.5, E5.5 and E6.5, respectively. Paired gonads were stored in a refrigerator at −80 °C. Additionally, a piece of extragonadal tissue from each embryo tissue was obtained in the meantime, for the subsequent sexing experiments.

### 4.2. Sexing and RNA Extraction

The DNA extraction of extragonadal tissues was individually performed using an animal tissue DNA extraction kit (Lifefeng, Shanghai, China) and sexing was performed using a PCR Taq master mix (Vazyme, Nanjing, China) with a primer of the sex-related gene *CHD1* [44] and the control gene *ATCB* (β-actin). The sequences of the primers were as follows:

CHD1-F: 5′-ATCTACCACTTTT CTCACGG-3′;

CHD1-R: 5′-TTCAGAGTGATAACGCATGG-3′;

β-actin-F: 5′-TGG ATG ATG ATA TTG CTG C-3′;

β-actin-R: 5′-ATC TTCTCC ATA TCA TCC C-3′.

Using CHD1 as the sexing primers, the presence of two bands (400–500 bp and 300–400 bp) on the gel electrophoresis image indicated female embryos, while the presence of a single band (400–500 bp) indicated male embryos. After sexing, homolateral gonads of the same stage and sex were pooled (with a total of 12 pools), and total RNA was isolated using Trizol Reagent (Thermo Scientific, Waltham, MA, USA). RNA purity (OD260/280) was measured using a NanoDrop spectrophotometer (Thermo Scientific, Waltham, MA, USA).

### 4.3. Library Construction and Transcriptome Sequencing

In PacBio sequencing, the total RNA of E4.5 gonads was reverse-transcribed to cDNA using SMARTer PCR cDNA Synthesis Kit (Clontech, Mountain View, CA, USA). After PCR amplification, BluePippin was used to select long-length fragments. Library construction was conducted using SMRTbell™ Template Prep Kit 1.0 (Pacific Biosciences, Menlo Park, CA, USA), and then was sequenced using Sequel™ Sequencing Kit 2.0. During Illumina sequencing, mRNA was separately purified from the total RNA of E4.5, E5.5, and E6.5 gonads using poly-T oligo-attached magnetic beads. cDNA was synthesized using random oligonucleotides and Super Script II for the first strand, and DNA Polymerase I and RNase H for the second strand. After the adenylation of the 3′ ends of the DNA fragments, Illumina PE adapter oligonucleotides were ligated. Fragments were purified using the AMPure XP system (Beckman Coulter, Brea, CA, USA) for selecting 400–500 bp of cDNA, then PCR-amplified using Illumina PCR Primer Cocktail in a 15-cycle PCR reaction and quantified using the Bioanalyzer 2100 system (Agilent, Santa Clara, CA, USA). The sequencing library was sequenced on Illumina NovaSeq 6000 platform. All transcriptome sequencing was performed by Shanghai Personal Biotechnology Cp. Ltd. (Shanghai, China).

### 4.4. Data Filtering and Alignment

The reference chicken genome was the *Gallus gallus* reference assembly GRCg6a. Raw data (fastq file) from Illumina sequencing were generated using the software of the sequencing platform, the Cutadapt (v1.15) software [45], to filter the sequencing data to obtain high-quality (HQ) sequences (clean data). Clean reads were mapped to the reference genome via HISAT2 v2.0.5 [46], and transcript expression was calculated via HTSeq v0.6.1 [47]; then, we used Fragments Per Kilobase of exon model per mMillion mapped fragments (FPKM) to standardize the expression. Raw data (bam file) from PacBio sequencing was generated using the software of the sequencing platform. Subreads corrected from zero-mode waveguide (ZMW) holes yielded RoI. RoI sequences were distinguished based on the sequencing adapters at both ends and the polyA tail sequences at the termini, resulting in full-length unspliced sequences, subsequently obtaining high-quality isoforms via clustering and polishing using IsoSeq3. The high-quality isoform was clustered using collapse_isoforms_by_sam.py of cDNA_Cupcake, and merged with chain_samples.py. The total full-length transcriptome was aligned with the reference genome using SQANTI2 [48], annotating and comparing with NR database using DIAMOND, using FPKM to standardize the expression. All raw RNA-seq data have been uploaded to CNCB (PRJCA019270).

### 4.5. Differentially Expressed Gene Analysis

The differentially expressed genes were analyzed using the DESeq2 R package [49]. The criteria for identifying DEGs were *p*-value < 0.05 and |log_2_ foldchange| > 1. We used the R language Pheatmap (1.0.12) software package [50] to perform a bi-directional clustering analysis of all different genes of samples. The generation of the heatmap involved calculating distances using the Euclidean method based on the expression levels of the same gene across different samples and the expression patterns of different genes within the same sample, followed by clustering using the complete linkage method. The sequencing results alignment scheme is detailed in Table 3 below.

### 4.6. Gene Ontology (GO) and Pathway Analysis

All DEGs were mapped to the terms of the GO database, and we calculated the numbers of differentially enriched genes in each term. GO enrichment analysis was performed using topGO for DEGs from Illumina and eggNOG-mapper for DEGs from PacBio [51]. The *p*-value was calculated via a hypergeometric test to confirm the main biological functions of the DEGs. GO terms with a *p*-value of <0.05 were considered significantly enriched.

### 4.7. Quantitative Real-Time PCR (qRT-PCR)

Additional 300 paired chicken embryos gonads at E4.5, E5.5 and E6.5 were collected separately for verifying the DEGs of males/females and left/right gonads via qPCR. After sex identification, about 30 homolateral gonads were pooled for each biological replicate (left and right; male and female; three stages) and RNA was extracted using Fast Pure Cell/Tissue Total RNA Isolation Kit (Vazyme) in accordance with the manufacturer’s instructions.

qRT-PCR analysis was performed using the CFX Connect Real-Time PCR detection system (Bio-Rad Laboratories, Hercules, CA, USA) and ChamQ Universal SYBR qPCR Master Mix (Vazyme, Nanjing, China). The primer sequences are listed in Table S25. The cycling parameters were as follows: 95 °C for 30 s, 40 cycles of 95 °C for 10 s, and 60 °C for 30 s, and then, 95 °C for 15 s, 60 °C for 60 s, and 95 °C for 15 s. The expression levels of genes were calculated relative to the expression of *β-actin* using the 2^−∆∆Ct^ method [52] and graphed using GraphPad Prism v8.3.0.538.

## 5. Conclusions

This study investigated sex-biased gene expression and dynamic transcriptome changes during chicken sex differentiation. PacBio and Illumina sequencing platforms were used to sequence gonadal transcriptomes at different developmental stages. The analysis revealed the significant differential expression of genes between males and females, with sex chromosomes playing a crucial role. As gonads differentiate, autosomal genes also contribute to sex-specific expression. Enrichment analysis highlighted the genes involved in DNA, RNA, and protein synthesis, as well as pathways related to sex determination and gonad development. Left-right asymmetric gene expression was also explored. Overall, the study provides insights into the molecular mechanisms underlying avian sex determination.

## Figures and Tables

**Table 1:** Partial list of DEGs and transcript isoforms of males and females at E4.5 in embryonic chicken gonads, as revealed via PacBio sequencing.

Gene Name	Transcript ID	F:fpkm	M:fpkm	FC: M/F	*p*-Value	Gene Location
*CETN3*	ENSGALT00000023609	0	60.7	Inf	0.00	Z(+)59940636-59957144
*POLR1D*	novel	0	84.83	Inf	0.00	4(−)11170879-11177016
*RPL34*	ENSGALT00000085406	0	146.21	Inf	0.00	4(−)37708386-37711694
*NDUFV2*	ENSGALT00000071937	0.02	94.36	Inf	0.00	2(−)98648739-98662576
*SEC62*	ENSGALT00000015276	0.04	20.32	621.52	0.00	9(−)20031613-20047157
*EMC2*	ENSGALT00000101212	0.17	69.67	379.42	0.00	2(+)132291685-132326563
*ISCA1*	ENSGALT00000020582	0.18	29.01	158.32	0.00	Z(−)40832095-40838966
*HMGB3*	ENSGALT00000014763	0.64	94.11	149.77	0.00	4(−)17645770-17651412
*IDH3A*	ENSGALT00000087067	2.05	51.19	24.97	0.00	10(−)4228399-4240203
*EIF4E*	ENSGALT00000051025	4.24	67.17	15.78	0.00	4(−)59890563-59909041
*REEP5*	ENSGALT00000089219	6.55	60.7	9.28	0.00	Z(+)46073109-46090480
*HMGN4*	ENSGALT00000056521	16.03	70.96	4.39	0.00	23(−)269675-272833
*PSMB7*	ENSGALT00000001657	139.01	68.59	0.49	0.00	17(−)9673940-9693735
*SNRPB2*	ENSGALT00000044185	140.25	51.2	0.36	0.00	3(+)5652610-5658848
*HMGB3*	ENSGALT00000014763	136.13	44.6	0.33	0.00	4(−)17645769-17651424
*ENO1*	ENSGALT00000089190	575.01	178.4	0.31	0.00	21(−)3220610-3233013
*POLR1D*	Novel	113.88	18.75	0.16	0.00	4(−)11170880-11177013
*-*	Novel	180.1	9.41	0.05	0.00	W(+)2134880-2136766
*ATP5F1AW*	ENSGALT00000088356	215.12	9.38	0.04	0.00	W(+)1391550-1415936
*HINTW*	ENSGALT00000051715	2508.64	105.82	0.04	0.00	W(+)1840711-1843135
*HINTW*	novel	158.03	5.86	0.04	0.00	W(+)1840727-1843634
*HINTW*	novel	178.42	5.35	0.03	0.00	W(+)1840716-1843899
*LOC107049046*	ENSGALT00000058877	736.13	24.84	0.03	0.00	W(+)2403711-2407146
*RPS25*	ENSGALT00000012476	176.43	0	0	0.00	24(−)5664049-5665730

F: female; M: male; Chr: chromosome; FC: foldchange; fpkm: Fragments Per Kilobase of exon model per Million mapped fragments.

**Table 2:** Partial sex-biased genes with relatively high expression and foldchange levels in embryonic chicken gonads, revealed via Illumina sequencing.

Gene Name	Gene ID	Chr	E4.5 Gonads				E5.5 Gonads				E6.5 Gonads			
			F:fpkm	M:fpkm	FC: M/F	*p*-Value	F:fpkm	M:fpkm	FC: M/F	*p*-Value	F:fpkm	M:fpkm	FC: M/F	*p*-Value
*HINTW*	ENSGALG00000035998	W	**590.99**	**24.23**	**0.04**	**0.00**	**445.06**	**7.12**	**0.02**	**0.00**	**338.51**	**1.26**	**0.00**	**0.00**
*LOC107049046*	ENSGALG00000040263	W	**185.19**	**5.84**	**0.03**	**0.00**	**171.05**	**1.95**	**0.01**	**0.00**	**159.39**	**0.24**	**0.00**	**0.00**
*HNRNPKL*	ENSGALG00000040086	W	**142.17**	**3.67**	**0.03**	**0.00**	**177.48**	**1.01**	**0.01**	**0.00**	**179.81**	**0.08**	**0.00**	**0.00**
*ATP5F1AW*	ENSGALG00000043758	W	**99.09**	**2.87**	**0.03**	**0.00**	**131.88**	**0.61**	**0.00**	**0.00**	**154.00**	**0.10**	**0.00**	**0.00**
*UBE2R2L*	ENSGALG00000048542	W	**28.17**	**0.65**	**0.02**	**0.00**	**38.85**	**0.17**	**0.00**	**0.00**	**42.17**	**0.02**	**0.00**	**0.00**
*SPIN1W*	ENSGALG00000040704	W	**16.67**	**0.46**	**0.03**	**0.00**	**27.25**	**0.09**	**0.00**	**0.00**	**33.04**	**0.04**	**0.00**	**0.00**
*UBAP2*	ENSGALG00000040780	W	**9.62**	**0.23**	**0.02**	**0.00**	**11.05**	**0.07**	**0.01**	**0.00**	**17.55**	**0.00**	**0.00**	**0.00**
*SMAD7B*	ENSGALG00000029545	W	**4.56**	**0.10**	**0.02**	**0.00**	**5.90**	**0.06**	**0.01**	**0.00**	**8.82**	**0.00**	**0.00**	**0.00**
*CYP19A1*	ENSGALG00000013294	10	0.02	0.03	1.70	0.95	0.13	0	0	0.29	**229.02**	**0.03**	**0.00**	**0.00**
*FOXL2*	ENSGALG00000029282	9	0.34	0.61	1.79	0.48	3.46	0.33	0.09	0.00	**17.16**	**0.45**	**0.03**	**0.00**
*FSHR*	ENSGALG00000009100	3	0.74	0.77	1.04	0.90	1.89	1.23	0.64	0.12	**30.72**	**2.17**	**0.07**	**0.00**
*ATPIF1*	ENSGALG00000038672	23	742.38	643.27	0.86	0.46	**328.95**	**140.52**	**0.42**	**0.00**	140.52	179.86	1.19	0.48
*TDH*	ENSGALG00000016651	3	52.71	55.59	1.05	0.60	62.04	53.17	0.84	0.35	**139.97**	**30.50**	**0.23**	**0.00**
*PISD*	ENSGALG00000006872	15	22.71	23.11	1.02	0.86	32.68	36.80	1.11	0.47	**114.20**	**36.59**	**0.33**	**0.00**
*GXYLT2*	ENSGALG00000007804	12	34.73	35.23	1.02	0.95	44.38	44.12	0.98	0.84	**113.08**	**42.62**	**0.39**	**0.00**
*SOX9*	ENSGALG00000004386	18	2.52	3.24	1.27	0.42	1.38	2.02	1.44	0.59	**1.51**	**4.58**	**3.15**	**0.00**
*AMH*	ENSGALG00000036346	28	0.54	0.99	1.86	0.14	**8.18**	**50.98**	**6.14**	**0.00**	**131.06**	**945.91**	**7.55**	**0.00**
*DMRT1*	ENSGALG00000010160	Z	27.89	45.88	1.66	0.00	65.15	122.66	1.85	0.00	**65.63**	**167.66**	**2.66**	**0.00**
*ZFAND5*	ENSGALG00000015144	Z	**59.77**	**119.55**	**2.01**	**0.00**	52.50	82.33	1.54	0.19	53.93	58.84	1.13	0.49
*SMAD2Z*	ENSGALG00000036001	Z	**12.96**	**26.98**	**2.08**	**0.00**	**17.61**	**39.04**	**2.18**	**0.00**	**25.20**	37.35	1.53	0.01
*PRLR*	ENSGALG00000003446	Z	**0.15**	**0.71**	**4.84**	**0.00**	**1.47**	**5.10**	**3.42**	**0.00**	**8.74**	**22.65**	**2.67**	**0.00**
*SMC2*	ENSGALG00000015691	Z	87.04	163.27	1.88	0.00	**108.77**	**224.85**	**2.03**	**0.00**	123.64	205.75	1.72	0.00
*HSDL2*	ENSGALG00000015663	Z	108.36	197.35	1.82	0.00	160.49	283.46	1.74	0.00	**175.57**	**339.56**	**2.01**	**0.00**
*CLTA*	ENSGALG00000015326	Z	152.30	268.87	1.76	0.00	147.40	250.55	1.67	0.00	**165.76**	**393.64**	**2.47**	**0.00**
*TAX1BP3*	ENSGALG00000004618	19	132.53	135.62	1.02	0.79	139.17	183.24	1.30	0.03	**143.78**	**340.41**	**2.46**	**0.00**
*HSPA5*	ENSGALG00000001000	17	126.60	137.33	1.09	0.38	147.36	203.10	1.36	0.01	**179.84**	**348.97**	**2.02**	**0.00**
*RPL17*	ENSGALG00000036774	Z	865.21	1518.39	1.75	0.00	667.40	1056.16	1.56	0.00	**646.65**	**1315.70**	**2.11**	**0.00**
*RPS6*	ENSGALG00000015082	Z	2416.42	4163.01	1.72	0.00	2147.00	3052.78	1.40	0.00	**1866.34**	**3788.96**	**2.11**	**0.01**
*RPL22L1*	ENSGALG00000009312	9	726.94	712.95	0.98	0.82	562.73	463.01	0.81	0.08	**469.58**	**1041.19**	**2.31**	**0.05**
*RPS23*	ENSGALG00000015617	Z	1715.83	2843.80	1.65	0.00	1093.88	1395.79	1.26	0.05	**783.93**	**1605.17**	**2.13**	**0.05**

F: female; M: male; Chr: chromosome; FC: foldchange (FC in bold font is significantly differentially expressed); fpkm: Fragments Per Kilobase of exon model per Million mapped fragments.

**Table 3:** Alignment scheme of sequencing results.

Sequence Technology	Comparison Content	Incubation Day	Gonad Sample
Illumina	DEG of male and female gonads	E4.5	Female vs. Male
		E5.5	Female vs. Male
		E6.5	Female vs. Male
	DEG of left and right gonads	E4.5	Female left vs. Female right
			Male left vs. Male right
		E5.5	Female left vs. Female right
			Male left vs. Male right
		E6.5	Female left vs. Female right
			Male left vs. Male right
PacBio	DEG of male and female gonads	E4.5	Female vs. Male

## Data Availability

The data presented in this study are openly available in China National Center for Bioinformation, reference number PRJCA019270.

## Supplementary Materials

The following supporting information can be downloaded at https://www.mdpi.com/article/10.3390/ijms241914597/s1.
